# 

**Published:** 1855-09

**Authors:** 


### RECEIPTS FOR THE MONTH OF AUGUST.

ILLINOIS.

Drs. J. G. Brown $2; A. G. Randall, $2; A. M. Abbott, $2; M. Davis, $2; John Garrison, $2; A. G. Porter, $2; C. M. Robertson, $2; T. S. Mills, $2; J. G. Tilden, $2; Wm. Hanly, $2.

WISCONSIN.

E. G. Dyer, $2; Rice & Clark, $2.

INDIANA.

Drs. G. A. Armstrong, $2; F. M. Denny, $2.

IOWA

Drs. Ephraim Clifford, $2; J. Doron, $2;

